# Effects of a new continuous nursing program on the short-term and long-term low back pain in patients after UBED: a retrospective study based on 282 patients

**DOI:** 10.3389/fsurg.2024.1443231

**Published:** 2024-08-29

**Authors:** Jucai Li, Yanli Song, Lumei Wu, Dan Su, Lin-Feng Wang

**Affiliations:** Department of Spine Surgery, Third Hospital of Hebei Medical University, Shijiazhuang, China

**Keywords:** UBE surgery, continuous nursing, low back pain, lumbar disc herniation, rehabilitation training

## Abstract

**Background:**

Unilateral biportal endoscopic discectomy (UBED) is a widely accepted minimally invasive surgery for the treatment of lumbar degenerative diseases. However, some patients continue to have persistent low back pain (LBP) symptoms in the short and long term after surgery, which may be related to improper postoperative nursing and rehabilitation of patients. Further research is needed to determine whether continuous nursing can improve the symptoms of patients after UBED.

**Methods:**

This study retrospectively enrolled 282 lumbar disc herniation (LDH) patients who underwent UBED in our hospital from January 2019 to January 2022. The patients were divided into two groups according to whether they accepted the continuous nursing program: 147 patients in the traditional nursing group and 135 patients in the continuous nursing group. Demographic characteristics, radiological parameters, and follow-up data of the patients were collected. Finally, the risk factors of LBP after UBED were analyzed.

**Results:**

The visual analog scale (VAS) score of LBP in the continuous nursing group was 0.97 ± 1.159 at 3 months and 0.61 ± 0.954 at 12 months after operation, and VAS of leg pain was 0.23 ± 0.421 at 12 months after operation, which were better than those in the traditional nursing group (1.51 ± 1.313, 1.10 ± 1.076, 0.68 ± 0.788, respectively, *p* < 0.001) The Oswestry disability index (ODI) score of the continuous nursing group was lower than that of the traditional nursing group at 12 months after operation (7.36 ± 6.526 vs. 12.43 ± 6.942, *p* < 0.001). The rehabilitation completion (7.98 ± 1.857), efficacy satisfaction (9.13 ± 1.101), and re-herniation worry scores (1.97 ± 1.217) in the continuous nursing group were better than those in the traditional nursing group (4.14 ± 3.066, 8.28 ± 1.240, 2.79 ± 1.973, respectively, *P* < 0.001). The re-herniation rate within 1 year was similar between the two groups (3/135 vs. 2/147, *p* = 0.673). No incision infection occurred. Multivariate regression analysis showed that risk factors for persistent LBP at 3-month follow-up were degenerative disc [odds ratio (OR): 2.144, CI: 1.306–3.519, *p* = 0.03], Pfirrmann grade (OR: 3.073, CI: 1.427–6.614, *p* = 0.04), and surgical time (OR: 0.969, CI: 0.937–1.003, *p* = 0.74). At the 12-month follow-up, the risk factors for persistent LBP were preoperative VAS of the legs (OR: 1.261, CI: 1.000–1.591, *p* = 0.05) and Pfirrmann grade (OR: 3.309, CI: 1.460–7.496, *p* = 0.04).

**Conclusion:**

Continuous nursing programs can improve the symptoms of short-term and long-term persistent LBP in patients after UBED, enhance the completion of rehabilitation training after UBED, alleviate patients' concerns about recurrence, and improve patients' satisfaction.

## Introduction

Lumbar disc herniation (LDH) is the main cause of low back pain (LBP) and leg pain. This pain is often severe and seriously affects the patient's work and quality of life (1). For patients with LDH who fail to respond to conservative treatment, surgery is often performed to remove part of the herniated intervertebral disc and relieve the compression of the lumbar plexus nerve root, thereby alleviating the pain symptoms of patients (2). To reduce the damage to soft tissue, various endoscopic techniques have been developed for spine surgery in recent years (3). Unilateral biportal endoscopic discectomy (UBED) is a representative minimally invasive surgery characterized by a short learning curve, sufficient decompression range, and low recurrence rate (4). The unilateral biportal endoscopic (UBE) technique involves creating two working channels for surgical manipulation through the gap between the multifidus muscle and the spinous process. One channel is inserted into the arthroscope to provide the operative field, and the other adjacent mini-incision channel is used for surgical manipulation to relieve nerve compression. It has been reported that patients with complete nerve root decompression have a 99.5% reduction in lower extremity pain symptoms (5).

However, after simple discectomy surgery, some patients experience persistent LBP, which seriously affects their postoperative rehabilitation progress and satisfaction (6). Although there were no separate reports on the rate of LBP relief or the probability of concurrent LBP after UBED, some studies have shown that the probability of persistent LBP after minimally invasive lumbar discectomy is approximately 8.4%–36.6% (7–9). The etiology of this type of persistent postoperative LBP is unclear. It is worth mentioning that there is a growing consensus that combining postoperative rehabilitation exercise with physical therapy, psychological intervention, and the use of painkillers can significantly improve treatment outcomes (10, 11). Early rehabilitation helps reduce muscle loss in the multifidus, erector spinae, and other lumbar back, enhance the stability of the lumbar spine (12), and promote the patients to return to normal life and work after minimally invasive lumbar surgery (13). However, due to social, family, economic, and other factors, patients are prone to some situations that are not conducive to the recovery of the disease in the postoperative recovery process. Issues such as poor compliance, inconsistent rehabilitation, failure to follow the step-by-step principle, and an incorrect understanding of the disease lead to poor long-term patient satisfaction after surgery.

Under the traditional nursing model, the hospitalization period for patients was only a few days, and the nursing care stopped on the day of discharge. However, the success of orthopedic surgery usually does not mean the end of treatment (14). Although our traditional model informs patients and their families that rehabilitation training should continue after discharge, the effectiveness of this post-discharge rehabilitation depends on the patient’s medical conditions and economic limitations. Only a few patients regularly follow the hospital rehabilitation guidance after discharge. In addition, the patient's psychological state can significantly influence postoperative rehabilitation. Negative attitudes and beliefs about chronic low back pain may lead to catastrophic thoughts and avoidance behaviors (15).

Continuous nursing is based on the rehabilitation characteristics of patients after discharge, through a variety of information means and personnel cooperation to establish intervention channels, so that patients can receive professional nursing intervention for a long time after discharge, promote rehabilitation, and reduce postoperative complications of patients (16). To improve the efficacy of UBED, our hospital designed a set of postoperative continuous nursing programs to guide and supervise the rehabilitation process of patients after UBED for 12 months. The purpose of this study is to investigate whether this continuous nursing program can improve the short-term and long-term LBP symptoms of patients after UBED and improve patient satisfaction.

## Methods

Patients who underwent UBED for lumbar disc herniation in our hospital from January 2019 to January 2022 were included in this study. Inclusion criteria included (a) patients with lumbar disc herniation who failed to respond to conservative treatment for more than 2 months and (b) complete medical records and imaging data were available. Exclusion criteria included (a) combined with ankylosing spondylitis, gluteal fasciitis, fractures, and other diseases leading to persistent LBP; (b) patients with previous lumbar surgery; (c) patients with cardiovascular and cerebrovascular diseases and poor physical conditions; and (d) deep vein thrombosis of the lower extremities, combined with peripheral neuropathy and other diseases affecting the patient's motor function. All patients received rehabilitation training guidance before discharge and chose whether to accept a postoperative transitional care program according to their wishes. The patients were followed up at 1, 3, 6, and 12 months after operation. A total of 312 patients met the criteria. Thirty-five of these patients were excluded because they withdrew from supervised training or did not complete follow-up. In this study, 147 patients were finally included in the traditional nursing group without receiving the transitional care scheme. One hundred and thirty-five patients received a complete continuing nursing program and were included in the continuing nursing group. Among them, two patients in the routine nursing group and three patients in the continuous nursing group experienced re-herniation and underwent revision surgery during the 1-year follow-up period, and their clinical efficacy was not included in the statistics, which was only used to compare the recurrence rate of patients between the two groups.

### Surgical procedure

After general anesthesia, the patient was placed in the prone position. A 5 mm entry point was cut at 1 cm above and below the point. The cephalic entry point was used as the observation port, and the caudal entry point was used as the operation port. After the blunt separation of the paraspinal muscles, a dilator was inserted to expand the channel. When the channels were satisfactory, the arthroscope was inserted into the viewing port. The next step is to look for the lower margin of the lamina of the upper vertebral body. After hemostasis, the soft tissue covering the bone and lamina space was removed. Part of the lamina and facet joints were then removed. The ligamentum flavum was carefully bitten and removed. After retracting the nerve root and exposing the disc, a discectomy was performed, and the annulus fibrosus was cauterized with bipolar electrocoagulation to cause it to shrink and form. Finally, the incision was sutured by hemostasis.

### The difference in nursing programs between the two groups

In the traditional nursing group, patients were informed of the matters to be attended to upon discharge, and a staged rehabilitation training program was informed and implemented by patients upon discharge. The web page description for rehabilitation was provided for patients to review anytime. The follow-up review was scheduled and informed by telephone. If the discomfort becomes worse, the frequency of follow-up can be increased.

In the continuous nursing group, the rehabilitation process after discharge was supervised and guided by a continuous nursing team consisting of specialist spinal nurses, spinal surgeons, and rehabilitation physicians. All patients joined the WeChat group and established real-time contact with the nursing team. The work content of the continuous nursing group was divided into three parts, including stage rehabilitation training, disease consultation, and psychological intervention.

1. Stage rehabilitation training: According to the time characteristics of disc recovery after minimally invasive discectomy surgery, the postoperative rehabilitation training of patients was divided into four stages: Stage 1, 0–2 weeks; Stage 2, 2–8 weeks; Stage 3, 8–16 weeks; and Stage 4, after 16 weeks. All patients entered the WeChat group at the corresponding period according to the rehabilitation stage. The rehabilitation training was supervised and guided by spinal specialist nurses and rehabilitation physicians in each chat group. The patients were supervised by the chat group, including video, characters, or voice, and each patient completed the appropriate rehabilitation training according to the stage missions and goals. Patients in the group spoke freely, communicated with each other, and answered and comforted other patients' questions. Missions and goals were established in Table 1.
2. Disease consultation: Provide consultation services and drug guidance for patients with LBP and leg pain during the rehabilitation process and timely answer the patient's doubts about the disease.
3. Psychological intervention: Encourage patients to carry out rehabilitation training. Provide guidance on postoperative living inconveniences. Organize patient exchange activities every month to share treatment and rehabilitation experience. Provide psychological counseling related to illness.

**Table 1:** Stage rehabilitation goal and mission.

Stage	Goal	Exercise mission
1	Prevent blood clots and muscle atrophy	1. Complete active and passive lower limb movements in bed. 2. Perform essential activities of daily living (e.g., eating, drinking, and toileting) while wearing a medical lumbar support belt
2	Regain walking function	1. Increase short-distance walking and raise the maximum daily walking distance each week. The upper limit for a single walk has been gradually increased from 200 m to 1 km. 2. Add upper extremity exercise
3	1. Increase the strength of the core muscles of the lumbar and abdomen. 2. Resume general work. Relieve the dependence on the lumbar support medical belt	1. Plank support, glute bridge, and alternating bird dog. Three groups a day, each group 10–30 times; 2. Non-manual workers gradually returned to general work. 3. The duration of lumbar support medical belt wear was gradually reduced until the dependence was completely relieved
4	1. Return life to normal. 2. Increase physical activity	1. Patients can do jogging, swimming, and other sports and avoid sports like badminton and high jumping. 2. Avoid weight bearing

This table shows the staged rehabilitation goals and exercise mission we established for patients after UBE.

Stage 1, 0–2 weeks; Stage 2, 2–8 weeks; Stage 3, 8–16 weeks; Stage 4, after 16 weeks.

### Data collection

Data collection included three parts: demographic characteristics, radiological parameters, and follow-up changes. (1) Demographic characteristics: We collected the information of patients by reviewing the medical records, including age, gender, body mass index (BMI), smoking or drinking status, hypertension, diabetes, preoperative visual analog scale (VAS) pain scores of low back and leg, preoperative Oswestry disability index (ODI) scores, surgical level, surgical times, and estimated blood loss. (2) Radiological parameters: These included the number of degenerative changes disc (Pfirrmann grade >2), Pfirrmann grade (disc of herniation) (17), Modic change (18), fatty infiltration of the paravertebral muscles (19), edema of lumbodorsal fascia in MRI, and facet joint preservation rate (20). (3) Follow-up changes: Length of stay, VAS scores of LBP, and leg pain were evaluated at 3 months and 12 months after operation, ODI score at 12 months after operation, and re-herniation at 12 months after operation. At the 12-month follow-up, we set three self-estimated scores, including rehabilitation completion score, efficacy satisfaction score, and re-herniation worry score, which was used to assess the degree of patient approval of the surgical effect. The maximum score for each item is 10 points. The rehabilitation completion score is the patient's self-estimated of the completion of rehabilitation training. Efficacy satisfaction score is a self-evaluation of the efficacy of surgery and the improvement of symptoms within 1 year. The re-herniation worry score reflected the worry grade about the recurrence of lumbar disc herniation. Unlike the previous two scores, higher scores on this scale indicate more worry. Finally, we used logistic regression to analyze the risk factors of patients with persistent LBP (VAS >3) at 3 and 12 months after surgery. Variables including age, gender, BMI, and preoperative VAS pain scores of the low back and legs, number of degenerative changes disc, Pfirrmann grade, Modic change, fatty infiltration of the paravertebral muscles, edema of lumbodorsal fascia, and facet joint preservation rate were included in the analysis.

### Statistical analysis

Statistical analysis was performed using SPSS 23.0 for Windows (IBM, Armonk, NY, USA). The continuous variables in the two groups were analyzed with the use of a one-way analysis of variance, and the results are expressed as means ± standard deviations. The chi-square test was used to compare the count data. *p* < 0.05 was considered statistically significant. Finally, the risk factors model of LBP was established by backward stepwise regression.

## Result

A total of 282 patients were included in this study. Five of these patients experienced re-herniation within 1 year, and their clinical results were used only to compare the re-herniation rate between the two groups. There were 145 patients in the traditional nursing group and 132 patients in the continuous nursing group, whose clinical outcomes were included in the statistical analysis. The average age was 44.4 ± 16.86 years in the traditional nursing group and 42.1 ± 12.76 years in the continuous nursing group. There were no significant differences in age, gender, BMI, and prevalence of underlying diseases between the two groups. Preoperative assessment of symptoms was similar in the two groups. Preoperative VAS (back) was 4.25 ± 1.516 in the traditional nursing group and 4.03 ± 1.446 in the continuous nursing group (*p* = 0.223). Preoperative VAS (leg) and preoperative ODI also had no statistical difference. Surgical records, including surgical level, surgical time, and estimated blood loss, were not significantly different (Table 2).

**Table 2:** Demographic characteristics.

	Traditional nursing (*n* = 145)	Continuous nursing (*n* = 132)	*p*
Age	44.4 ± 16.86	42.1 ± 12.76	0.197
Female/male	58/87	45/87	0.309
BMI	25.32 ± 3.906	25.51 ± 4.085	0.698
Hypertension (*n*)	20	21	0.620
Diabetes (*n*)	10	13	0.374
Drinking (*n*)	36	36	0.643
Smoking (*n*)	33	29	0.875
Preoperative VAS (back)	4.25 ± 1.516	4.03 ± 1.446	0.223
Preoperative VAS (leg)	6.99 ± 1.585	6.68 ± 2.054	0.166
Preoperative ODI	65.23 ± 6.706	64.41 ± 9.521	0.403
Surgical level			0.230
L3/4	11	4	
L4/5	64	63	
L5/S1	65	65	
Surgical time (min)	62.8 ± 10.94	64.4 ± 12.15	0.262
Estimated blood loss			0.832
<50 ml	96	89	
≥50 ml	49	43	

Values are presented as means and standard deviations or number.

BMI, body mass index; VAS, visual analog scale; ODI, Oswestry disability index.

### Radiological parameters

There was no difference between the two groups in the number of degenerate discs, Pfirrmann grades, Modic changes, fatty infiltration of muscle, or edema of lumbodorsal fascia. Lumbar CT after surgery showed that the facet joint preservation rate was similar between the two groups (Table 3).

**Table 3:** Radiological parameters difference between the two groups.

	Traditional nursing	Continuous nursing	*p*
Degenerative disc (*n*)			0.354
*n* = 1	76	82	
*n* = 2	55	42	
*n* = 3	44	7	
*n* = 4	3	1	
Pfirrmann grade			0.383
Grade 3	4	5	
Grade 4	110	107	
Grade 5	31	20	
Modic change			0.945
0	119	110	
Type 1	9	7	
Type 2	12	10	
Type 3	4	5	
Fatty infiltration of muscle			0.705
<10%	122	106	
10%–50%	15	17	
>50%	8	9	
Edema of lumbodorsal fascia	11	10	0.997
Facet joint preservation			0.890
<90%	68	63	
≥90%	77	69	

### Difference in postoperative variables between the two groups

Table 4 presents the VAS changes and patients' satisfaction after surgery. There was no significant difference in the length of stay in the hospital between the two groups. However, at the 3-month follow-up, the VAS score of LBP in the traditional nursing group (1.51 ± 1.313) was higher than that in the continuous nursing group (0.97 ± 1.159) ( *p* < 0.001). VAS of leg pain relief was similar between the two groups (*p* = 0.689). At the 12^th^-month follow-up, there were significant differences in symptom relief assessment indexes between the two groups. The VAS score of LBP was 1.10 ± 1.076 in the traditional nursing group and 0.61 ± 0.954 in the continuous nursing group. The VAS score of leg pain was 0.68 ± 0.788 in the traditional nursing group and 0.23 ± 0.421 in the continuous nursing group. When the patient’s daily activity function was assessed, the ODI score was different between the two groups (12.43 ± 6.942 vs. 7.36 ± 6.526, *p* < 0.001). In terms of patient self-estimated scores, there were significant differences between the two groups. The rehabilitation completion score was 4.14 ± 3.066 in the traditional nursing group and 7.98 ± 1.857 in the continuous nursing group, and the efficacy satisfaction score was 8.28 ± 1.240 in the traditional nursing group and 9.13 ± 1.101 in the continuous nursing group. The re-herniation worry score was 2.79 ± 1.973 in the traditional nursing group and 1.97 ± 1.217 in the continuous nursing group. There was no significant difference in the re-herniation rate between the two groups. The re-herniation rate was 2/147 in the traditional nursing group and 3/135 in the continuous nursing group, both of which were kept at a low level, and there was no difference between the two groups.

**Table 4:** Follow-up result of VAS changes, ODI, and self-estimated scores.

	Traditional nursing	Continuous nursing	*p*
Length of stay (day)	5.71 ± 1.178	5.66 ± 1.497	0.197
VAS-back 3rd month	1.51 ± 1.313	0.97 ± 1.159	<0.001
VAS-leg 3rd month	0.41 ± 0.534	0.43 ± 0.497	0.689
VAS-back 12th month	1.10 ± 1.076	0.61 ± 0.954	<0.001
VAS-leg 12th month	0.68 ± 0.788	0.23 ± 0.421	<0.001
ODI 12th month (%)	12.43 ± 6.942	7.36 ± 6.526	<0.001
Rehabilitation completion score	4.14 ± 3.066	7.98 ± 1.857	<0.001
Efficacy satisfaction score	8.28 ± 1.240	9.13 ± 1.101	<0.001
Re-herniation worry score	2.79 ± 1.973	1.97 ± 1.217	<0.001
Re-herniation (*n*)	2 (1.36%)	3 (2.27%)	0.673
Infection	0	0	–

This table shows the follow-up results of patients at different stages after UBE surgery. The *X* month following the VAS score indicates the time of postoperative follow-up, and all other scoring endpoints are the 12th month after surgery.

### Risk factors analysis of postoperative persistent LBP

Logistic regression analysis (Tables 5, 6) showed that the risk factors of persistent LBP at 3 months (short-term) were the number of degenerative discs [odds ratio (OR): 2.144, CI: 1.306–3.519, *p* = 0.03], Pfirrmann grade (OR: 3.073, CI: 1.427–6.614, *p* = 0.04), and surgical time (OR: 0.969, CI: 0.937–1.003, *p* = 0.74). During the 12-month long-term follow-up, the risk factors for LBP were preoperative VAS (legs) (OR: 1.261, CI: 1.000–1.591, *p* = 0.05) and Pfirrmann grade (OR: 3.309, CI: 1.460–7.496, *p* = 0.04).

**Table 5:** Risk factors for short-term persistent LBP.

	Odds ratio	95% CI	*p*
Degenerative disc (*n*)	2.144	1.306–3.519	0.03
Pfirrmann grade	3.073	1.427–6.614	0.04
Surgical time	0.969	0.937–1.003	0.74

**Table 6:** Risk factors for long-lasting LBP.

	Odds ratio	95%CI	*p*
Preoperative VAS (leg)	1.261	1.000–1.591	0.05
Pfirrmann grade	3.309	1.460–7.496	0.04

## Discussion

The results of this study show that continuous nursing can improve the short-term and long-term LBP symptoms of patients after UBED. In addition, at the 12th-month follow-up, the continuous nursing group showed better activity function, with an ODI score of 7.36 ± 6.526, which was significantly lower than that of the traditional nursing group (4.14 ± 3.066). Through a series of methods of continuous nursing, we significantly increased the completion degree of rehabilitation after surgery, improved the compliance of patients, and improved long-term satisfaction (Table 4).

Step therapy for LDH is the accepted treatment strategy at present, and single discectomy is the median plan of step therapy, which is suitable for most patients with LDH (21). However, recurrent LBP can occur in 25%–36.6% of patients after minimally invasive lumbar discectomy (9, 22). LBP seriously affects patients’ normal work and quality of life, exacerbates the use of painkillers (23), and reduces patient satisfaction with surgery and efficacy (24). There are many reasons for chronic LBP in patients after lumbar surgery. According to the difference of source tissue, it can be divided into intervertebral disc source, paravertebral muscle source, and ligament injury. Multivariate analysis suggested that chronic LBP may be associated with disc height reduction, re-herniation, Modic changes, fat infiltration of paravertebral muscle, lumbar dorsal fascia edema, and lumbar facet joint osteoarthritis (5, 25). We suggest that a longer operation time may indicate that the patient is obese or that there are difficulties in the operation, which can cause more severe muscle damage and lead to longer back pain in the short term (Table 5). Although removal of a herniated lumbar disc can significantly relieve symptoms of leg pain, there is limited improvement in symptoms of low back pain (26). There are still some difficulties in diagnosing the etiology of LBP. It is worth mentioning that sonography measures can explore the multifidus and erector spinae muscles dynamically and non-invasively. A study used ultrasound to investigate the thickness and changes of the multifidus and erector spinae muscles in sitting and lying positions for the prediction and diagnosis of pain etiology in patients with chronic low back pain (27). Improving LBP after lumbar surgery, improving lower limb function, and reducing postoperative complications are the main objectives of postoperative nursing (28).

Two important means of continuous nursing are supervised rehabilitation training and intervention of surface emotions. A large number of studies have shown that continuous nursing can improve the joint function of patients after knee replacement and hip replacement by enhancing postoperative rehabilitation training, the completion of rehabilitation training, and the joint function of patients after knee replacement and hip replacement (29, 30). In addition, the continuous nursing group can timely understand the psychological state of patients and carry out necessary interventions through a variety of means, relieve the anxiety and fear of patients, and effectively improve the quality of life of patients (31).

Rehabilitation training is the key method for patients after lumbar discectomy surgery. Postoperative rehabilitation training can improve blood circulation, prevent muscle atrophy, enhance strength, increase spinal stability, and improve quality of life (32). Afzal et al. (33) conducted a meta-analysis and found that postoperative rehabilitation training was recommended to start 1–2 months after surgery and last for 3 months and postoperative rehabilitation training could significantly improve patients' long-term pain scores. However, the study did not consider the effect of different surgical methods on spinal stability. The start time, duration, and exercise intensity of lumbar non-fusion postoperative rehabilitation are still not uniform and need to be further optimized. At present, there are many rehabilitation training programs after lumbar surgery, but most of them are training programs after lumbar fusion (34, 35). After fusion surgery, the muscle destruction is large, but the spinal stability is high, and the risk of recurrence is very low. Therefore, the rehabilitation training program after fusion mostly suggests that patients go to the ground early and gradually resume daily life. Unlike fusion surgery, after simple discectomy, the nucleus pulposus is partially preserved, and the annulus fibrosus has a longer healing period (36, 37). Those patients are characterized by minimal muscle destruction and better spinal stability, while the likelihood of re-herniation of the nucleus pulposus is higher (32). Although the incision of UBED healed well within 14 days after operation, many patients still maintained a worried attitude toward rehabilitation exercise because they were worried about re-herniation (38, 39). In the traditional nursing group, although each patient received good rehabilitation education and training before discharge, such a short period of training seems to be difficult to achieve satisfactory results.

The healing cycle of muscles, ligaments, and bones after orthopedic surgery is longer, and less or later rehabilitation training is often thought to lead to a poor prognosis, including local surgical pain and joint stiffness (40). Continuous nursing is thought to improve postoperative outcomes in orthopedic patients (41). Based on the characteristics of physiological structure recovery of patients after UBED, we established a series of staged rehabilitation training guidance in combination with rehabilitation medicine experts. However, in the previous outpatient follow-up process, it was found that the patient's compliance was lacking, it was difficult to consciously complete the appropriate rehabilitation training action, and there was resistance to rehabilitation training. Therefore, we set up an oversight group consisting of senior nurses, spine surgeons, and rehabilitation medicine physicians. Postoperative continuous nursing was provided to patients, rehabilitation training was supervised, psychological guidance and medication advice were provided outside the hospital, and a patient communication community was established to improve the outcome of patients after UBED. This study retrospectively analyzed the population who had previously participated in this program. It is found that continuous nursing can significantly improve postoperative persistent LBP and improve the quality of life of patients. Schwartz et al. (42) found that rehabilitation exercise after spinal surgery can reduce patients' anxiety and improve the recovery trajectory of patients after spinal surgery and recommended that patients exercise as early as possible and persist for a long time after spinal surgery. Recently, a study on the exercise mode of patients after percutaneous endoscopic lumbar discectomy found that although the training mode was designed according to the structure of the lumbar dynamic chain and the postoperative period did not increase the multifidus muscle of patients, it could promote the rehabilitation of patients and improve the activity score of patients at 6 months compared with the conventional lumbar and back muscle exercise (43). This new rehabilitation mode combines the chain movement mode of the waist, hip, and leg and provides a new idea for the rehabilitation mode of LDH in the future. Targeted therapeutic exercise can improve the long-term prognosis of patients with chronic neck pain and improve muscle and soft tissue injury (44).

It is important to note that persistent LBP is often associated with social and psychological factors. In the process of continuous nursing, we found that patients paid more attention to the condition consultation service and psychological counseling service. Fear-avoidance behaviors, kinesiophobia, and anxiety were common in patients after minimally invasive lumbar surgery. It will cause great interference to the rehabilitation process of patients. In encouraging the rehabilitation exercise of patients after lumbar surgery, the potential obstacles caused by psychological and social factors should also be alleviated (45). A previous study found that it was postoperative, rather than preoperative, fear beliefs about exercise that were associated with improved postoperative pain, disability, and quality of life. The researchers recommend screening patients for fear of exercise after cervical and lumbar surgery and incorporating cognitive behavioral techniques into postoperative rehabilitation of high-risk spinal patients (45). Another advantage, continuous nursing can reduce the economic burden of patients through family support (46).

Most of the traditional lumbar postoperative patients recover at home after discharge, and the wound healing, symptom recovery, and condition change all depend on outpatient review. A small number of patients go to community hospitals or rehabilitation hospitals for post-discharge rehabilitation treatment, which has high time and economic cost and low relative benefit. With the development of social information, the nursing mode after discharge has also been reformed. Professional nursing staff can continue nursing care with patients through various applications such as Wechat. The use of graphics, video, telephone communication, and other ways to answer patient concerns guides the rehabilitation process of patients and enriches the content of continued nursing (47, 48). It is especially beneficial for patients after orthopaedic surgery, who need to undergo a long-term rehabilitation process after discharge. High-quality popular science videos and articles can effectively increase patients' understanding of the disease, enhance patients' confidence in rehabilitation, and timely detect and intervene in poor prognosis. The Internet-based continuous nursing can simplify and enhance the medical care service after orthopedic surgery and achieve cost reduction and efficiency increase.

## Conclusion

This study describes a continuous nursing program for the lumbar spine after UBED, focusing on the three key issues of stage rehabilitation training, disease consultation, and psychological intervention after UBED and establishing a convenient contact channel to guide and help patients. Compared with the traditional post-discharge nursing model, continuous nursing effectively improved the short- and long-term LBP symptoms of patients after UBED, reduced the mobility disorders of patients, improved the satisfaction of patients with surgery, and did not increase the probability of re-herniation of patients. Risk factor analysis of LBP in UBED also showed that patients with more and more severe disc degeneration and severe lower extremity symptoms before surgery were more likely to have persistent LBP symptoms after surgery. This also suggests that nursing staff, for patients with such high-risk factors, should strengthen postoperative nursing and follow-up, try to improve patient symptoms, and improve patient satisfaction.

Based on the results of this study, we recommend continuing nursing for patients after minimally invasive lumbar surgery. Through WeChat, telephone, and other means of communication and supervision, patients received staged rehabilitation training (Table 1), to provide a long-term consulting platform and timely intervention in patients with negative emotions.

### Limitation

Our study has some limitations: (1) This study provided a model of continuous nursing after UBED. However, due to policy issues, the cost and effectiveness were not considered. (2) This study is a single-center retrospective study and only discusses patients after UBED. Further research is needed to verify the clinical significance of transitional care for patients after other LDH surgical methods and patients in different medical centers. (3) In this study, the use of the WeChat platform to clock in supervision can not effectively supervise the completion of the actual rehabilitation training of patients. (4) Although most of our results were statistically significant, we lacked an exploration of the minimal clinically important difference (MCID) of these results. Whether the clinical significance of continuous nursing can be further improved remains to be discussed.

## Data Availability

The raw data supporting the conclusions of this article will be made available by the authors, without undue reservation.
